# The EhCPADH112 Complex of *Entamoeba histolytica* Interacts with Tight Junction Proteins Occludin and Claudin-1 to Produce Epithelial Damage

**DOI:** 10.1371/journal.pone.0065100

**Published:** 2013-06-07

**Authors:** Abigail Betanzos, Rosario Javier-Reyna, Guillermina García-Rivera, Cecilia Bañuelos, Lorenza González-Mariscal, Michael Schnoor, Esther Orozco

**Affiliations:** 1 Department of Infectomics and Molecular Pathogenesis, Center for Research and Advanced Studies of the National Polytechnic Institute, Distrito Federal, Mexico; 2 Department of Physiology, Biophysics and Neuroscience, Center for Research and Advanced Studies of the National Polytechnic Institute, Distrito Federal, Mexico; 3 Department of Molecular Biomedicine, Center for Research and Advanced Studies of the National Polytechnic Institute, Distrito Federal, Mexico; 4 Institute of Science and Technology of the Federal District, Distrito Federal, Mexico; 5 Autonomous University of Mexico City, Distrito Federal, Mexico; University Hospital Hamburg-Eppendorf, Germany

## Abstract

*Entamoeba histolytica,* the protozoan responsible for human amoebiasis, causes between 30,000 and 100,000 deaths per year worldwide. Amoebiasis is characterized by intestinal epithelial damage provoking severe diarrhea. However, the molecular mechanisms by which this protozoan causes epithelial damage are poorly understood. Here, we studied the initial molecular interactions between the *E. histolytica* EhCPADH112 virulence complex and epithelial MDCK and Caco-2 cells. By confocal microscopy, we discovered that after contact with trophozoites or trophozoite extracts (TE), EhCPADH112 and proteins forming this complex (EhCP112 and EhADH112) co-localize with occludin and claudin-1 at tight junctions (TJ). Immunoprecipitation assays revealed interaction between EhCPADH112 and occludin, claudin-1, ZO-1 and ZO-2. Overlay assays confirmed an interaction of EhCP112 and EhADH112 with occludin and claudin-1, whereas only EhADH112 interacted also with ZO-2. We observed degradation of all mentioned TJ proteins after incubation with TE. Importantly, inhibiting proteolytic activity or blocking the complex with a specific antibody not only prevented TJ protein degradation but also epithelial barrier disruption. Furthermore, we discovered that TE treatment induces autophagy and apoptosis in MDCK cells that could contribute to the observed barrier disruption. Our results suggest a model in which epithelial damage caused by *E. histolytica* is initiated by the interaction of EhCP112 and EhADH112 with TJ proteins followed by their degradation. Disruption of TJs then induces increased paracellular permeability, thus facilitating the entry of more proteases and other parasite molecules leading eventually to tissue destruction.

## Introduction

*Entamoeba histolytica* is the protozoan responsible for human amoebiasis that infects 50 million people and kills between 30 and 100 thousand individuals per year around the world [1]. Invasive amoebiasis is characterized by disruption and invasion of the colonic mucosa by *E. histolytica* trophozoites resulting in colonic ulceration [2], [3], [4]. The concerted activity of *E. histolytica* proteins, like the EhCPADH112 complex [5], the Gal/GalNAc lectin [6], [7], amoebapores [8] and cysteine proteases [9], [10], [11], lyses enteric cells that are subsequently ingested by trophozoites [12].

The EhCPADH112 complex (124 kDa) is formed by the EhCP112 cysteine protease (50 kDa) and the EhADH112 adhesin (75 kDa) [5]. The complex, involved in adhesion, cytolysis and phagocytic activities of *E. histolytica,* is diminished in adherence- and virulence-deficient mutant trophozoites and is recognized by sera of patients with intestinal and hepatic *E. histolytica* infections [5], [13].

EhADH112 contains three putative transmembrane domains and a carboxy terminus adherence epitope which is recognized by the mαEhCPADH112 monoclonal antibody that, similarly to the EhADH243 recombinant polypeptide, inhibits target cell adherence and phagocytosis [5], [14]. At the amino terminal region, EhADH112 has a Bro1 domain and a consensus site for Src-tyrosine phosphorylation, both of which have been involved in signal transduction [15], [16].

On the other hand, EhCP112 is a papain-like protease formed by a signal peptide, a propeptide and a domain characterized by the catalytic triad CHN [5]. EhCP112 also contains a putative transmembrane segment [5], an ERFNIN motif, characteristic for cathepsins H- or L-like propeptides [17], [18], and a RGD sequence for interaction with integrins [5], [19]. An EhCP112 recombinant protein containing the pro-peptide and the mature enzyme digests gelatin, type I collagen, fibronectin and hemoglobin [11].

In epithelia, tight junctions (TJs) seal intercellular contacts avoiding luminal penetration by pathogens. TJs constitute a belt-like region between epithelial cells that separate the apical from the lateral plasma membrane and regulate the passage of ions and molecules through the paracellular pathway [20]. TJ strands are composed of membrane integral proteins such as TAMP (tight junction–associated MARVEL proteins, with occludin being the best studied member of this protein family), JAM (junctional adhesion molecules), and more than 20 members of the claudin family [21]. These proteins interact with the actin cytoskeleton via TJ adaptor proteins like (zonula occludens) ZOs, membrane-associated guanylate kinase inverted **(**MAGIs) and cingulin [21], [22], [23].

After contact with trophozoites, epithelial monolayers show a rapid decrease of transepithelial electrical resistance (TER), accompanied by an increase of paracellular permeability suggesting disturbance of TJs [12], [24], [25], [26], [27]. These changes are coupled to ZO-1 degradation, ZO-2 dephosphorylation and disassociation of ZO-1 from ZO-2 [26]. In addition, prostaglandin E_2_ (PGE_2_) secreted by *E. histolytica,* disassociates claudin-4 from TJs and increases paracellular permeability for sodium [27].

Here, we studied the role of EhCPADH112 in facilitating the entrance of *E. histolytica* trophozoites into the epithelium through the paracellular pathway. Our results show that EhCPADH112, EhCP112 and EhADH112 proteins are present at TJs and co-localize with occludin after incubating epithelial MDCK cells with trophozoite extracts (TE). Additionally, we demonstrate a differential interaction of EhCPADH112 complex and its components EhCP112 and EhADH112 with occludin, claudin-1, ZO-1 and ZO-2, and specific degradation of these TJ components by this complex. Our findings suggest that the EhCPADH112 complex mediates epithelial damage by interacting the with TJ proteins.

## Materials and Methods

### Cell Culture

*E. histolytica* trophozoites of strain HM1:IMSS clone A [28], were axenically cultured at 37°C in TYI-S-33 medium and harvested during logarithmic growth phase [29].

MDCK (Madin Darby canine kidney) type I and Caco-2 (human colonic adenocarcinoma) epithelial cells were grown in DMEM medium supplemented with penicillin (100 i.u./ml; In Vitro), streptomycin (100 mg/ml; In Vitro), 10% fetal bovine serum (Gibco) and insulin (0.08 U/ml; Eli Lilly), at 37°C and 5% CO_2_ [30].

### Antibodies

As primary antibodies we used rabbit polyclonal antibodies against EhCPADH112 (pαEhCPADH112) [13]; KYHSNSTYVQFYNHT, an EhCP112 specific polypeptide (pαEhCP112); EEVSLEKEPTESKG, an EhRabB specific polypeptide (pαEhRabB) [31]; claudin-1 (pαcl-1; Invitrogen), ZO-1 (pαZO-1; Invitrogen), ZO-2 (pαZO-2; Invitrogen), β-catenin (pαβ-catenin; Sigma-Aldrich) and MAP LC3β (pαLC3; Santa Cruz Biotechnology, Inc; kindly provided by Dr. Juan Kouri, Department of Infectomics and Molecular Pathogenesis, CINVESTAV-IPN). Monoclonal antibodies used were: EhCPADH112 (mαEhCPADH112) [13]; and glyceraldehyde-3-phosphate dehydrogenase (mαGAPDH; Chemicon). Rabbit polyclonal and mouse monoclonal antibodies against occludin were purchased from Invitrogen (pαocc and mαocc). A rat polyclonal antibody against 160 aa of the EhADH112 Bro1 domain (pαEhADH112) has been described previously [16]. Secondary antibodies derived from goat (Invitrogen) included: HRP-labelled anti-rabbit, -mouse and -rat IgGs; FITC-labelled anti-rabbit and -mouse IgGs; FITC-labelled anti-mouse IgM; Cy5-labelled anti-rabbit IgG; and TRITC-labelled anti-rat and anti-mouse IgGs.

### Secreted Products (SP) of *E. histolytica*

SP were prepared and collected as described previously [11]. Briefly, trophozoites were washed three times in PBS, and incubated for 2 h at 37°C under periodic swirling. Supernatants containing SP were collected by centrifugation at 2,000 rpm for 10 min and filtered through 0.22 mm cellulose acetate membranes (Millipore). Viability of trophozoites after incubation was ∼95%, as determined by trypan blue exclusion assay.

### Preparation of TE

Defined numbers of trophozoites were washed twice in PBS at 4°C and lysed by freeze-thawing in the presence of 20 mM PHMB (p-hydroxymercuribenzoate) as protease inhibitor [5].

### Preparation of MDCK Lysates

MDCK cells were washed with PBS, scraped with a rubber policeman and lysed for 30 min in RIPA buffer (40 mM Tris-HCl pH 7.6, 150 mM NaCl, 2 mM EDTA, 10% glycerol, 1% Triton X-100, 0.5% sodium deoxycholate, 0.2% SDS, 1 mM PMSF and Complete™ protease inhibitor cocktail) under continuous and vigorous shaking. Extracts were sonicated three times for 30 s and centrifuged for 15 min at 14,000 rpm to eliminate undissolved cellular debris [32].

### Interaction of Epithelial Cells with, Live Trophozoites (T), Trophozoite Extracts (TE), or Secreted Proteins (SP)

Confluent MDCK cell monolayers were incubated with T (an MDCK-to-ameba ratio of 1∶1), TE (an MDCK-to-ameba ratio of 1∶2) or SP (an MDCK-to-ameba ratio of 1∶10) at 37°C for the indicated times. Confluent Caco-2 cells were incubated with T (an Caco-2-to-ameba ratio of 4∶1), at 37°C for the indicated times. After interaction, epithelial cells were washed 5 times with PBS to eliminate unbound molecules or trophozoites. Additionally, in some experiments TE were pre-incubated for 20 min with protease inhibitors (Complete™ and 40 µg/ml E-64) or mαEhCPADH112 (300 µg/1×10^6^ trophozoites) [5].

### Transepithelial Electrical Resistance (TER)

Confluent MDCK and Caco-2 cells were grown on transwell filters (0.4 µm pore size, Corning) and incubated for 2.5 h with T, TE, SP or with TE pre-treated with protease inhibitors or mαEhCPADH112. TER values were obtained using an EVOM epithelial voltohmmeter (World Precision Instruments) and each transwell measurement was normalized accordingly to its initial value before treatment [30]. Three independent experiments were carried out in triplicate.

### Immunofluorescence Assays

MDCK cell monolayers incubated with T, TE or SP were fixed and permeabilized with 96% ethanol for 30 min at −20°C. Then, cells were blocked with 0.5% BSA and 0.03% saponin and incubated overnight with mαEhCPADH112 or a mixture of mαEhCPADH112 or pαEhCP112 with either pαocc, pαcl-1, pαβ-catenin or pαEhADH112 antibodies. Cells were washed and incubated with species-specific FITC-labelled, TRITC-labelled or Cy5-labelled secondary antibodies. In some experiments, actin was stained with TRITC-phalloidin and nuclei were labeled with DAPI for 5 min. After washing, cells were mounted using Vectashield (Vector) [32] and analysed by laser confocal microscopy (Leica TCS_SP5_MO).

### Immunoprecipitation Experiments

MDCK cells incubated for 20 min with TE were lysed with RIPA/HO buffer (1∶1 vol/vol; HO buffer: 50 mM HEPES pH 7.5, 150 mM NaCl, 1 mM EGTA, 1.5 mM MgCl_2_, 10% glycerol, 1% Triton X-100 and the Complete™ protease inhibitor cocktail) for 15 min at 4°C, incubated for 3 h with Protein G-Sepharose to clear proteins that would bind unspecifically (Invitrogen) and then incubated overnight at 4°C with the pαEhCPADH112 antibody or with rabbit pre-immune serum. Next, samples were mixed with recombinant Protein G-Sepharose for 3 h, washed five times with HO buffer, boiled with sample buffer and centrifuged for 15 min at 14,000 rpm. Supernatants were analysed by SDS-PAGE and Western blot [32].

### Western Blot

Protein samples were separated by 8%, 10%, 12% or 15% SDS-PAGE and transferred to nitrocellulose membranes. Membranes were incubated overnight with the indicated primary antibodies and, after washing, for 1 h with the corresponding peroxidase-labelled secondary antibodies. Protein bands were visualized by chemiluminescence on X-ray films [32]. Membranes were stained with Ponceau S Red staining solution (Sigma Aldrich) to control protein loading and transfer.

### Overlay Assays

An excess of TE (100 µg/lane) immobilized on a nitrocellulose membrane was incubated overnight at 4°C with MDCK cell extracts (200 µg/ml). After 10 washes with cold PBS for 5 min each, membranes were processed for Western blot assays, using pαcl-1, pαocc, pαZO-1, pαZO-2 or mαGAPDH antibodies. Alternatively, membranes with MDCK cell proteins were incubated overnight at 4°C with TE followed by pαEhADH112 or pαEhCP112 antibodies [33]. The same membranes with TE were stripped (stripping buffer: 100 mM β-mercaptoethanol, 2% SDS and 62.5 mM Tris-HCl pH 6.8) of MDCK cell extracts and antibodies, and developed with the pαEhCPADH112 antibody. Membranes containing MDCK cell proteins were washed, stripped and incubated with pαocc, pαcl-1, pαZO-1 or pαZO-2 antibodies. The membranes were stained with Ponceau S Red staining solution before and after incubation with lysates, in order to control protein loading and transfer (data not shown).

### Degradation Experiments

MDCK cell monolayers were incubated for 30 min with different amounts of TE (lysates from 10, 25, 50 and 100×10^3^ trophozoites) or with TE (from 100×10^3^ trophozoites) pre-treated with protease inhibitors or mαEhCPADH112 as described above. After extensive washes, epithelial cells were lysed and processed for Western blot assays. To detect occludin, claudin-1, ZO-1 and ZO-2, proteins were separated by 10%, 15%, 6% and 8% SDS-PAGE, respectively.

### Detection of Apoptosis and Necrosis

Translocation of phosphatidylserine (PS) to the external surface of the cell membrane and uptake of propidium iodide was determined using the Annexin V Apoptosis Detection Kit FITC (eBioscience), according to the manufacturer’s instructions [34]. MDCK cells were incubated with TE (1∶2 ratio) for 2, 10 and 30 min or treated for 4 h with 0.5 M staurosporine (Sigma-Aldrich) as a positive control for induction of apoptosis. The percentage of early apoptotic cells (positive for only annexin V), late apoptotic cells (positive for both annexin V and propidum iodide) and necrotic cells (positive only for propidium iodide) from a pool of 20,000 cells was determined using a fluorescence-activated cell sorting (FACS) Vantage SE BD system (BD Biosciences). Experiments were carried out in quadruplicate.

### Autophagy Assays

MDCK cell monolayers were incubated with TE (1∶2 ratio) for 2, 10 and 30 min or treated for 2 h with 10 µM rapamycin (Tocris Bioscience) as a positive control for autophagy [35]. Then, cells were immunofluorescently labelled using the pαLC3 antibody to detect the formation of autophagosomal vacuoles.

### Statistics

All data shown are representative of three independent experiments unless stated otherwise. GraphPad Prism 5 software was used for statistical analysis. Data were analysed by two-tailed Student *t*-test. Statistical significance was assumed at *P*<0.05. All results are displayed as mean with standard error of the mean.

## Results

### The *E. histolytica* EhCPADH112 Virulence Complex Induces Epithelial Barrier Disruption

**Figure 1:**
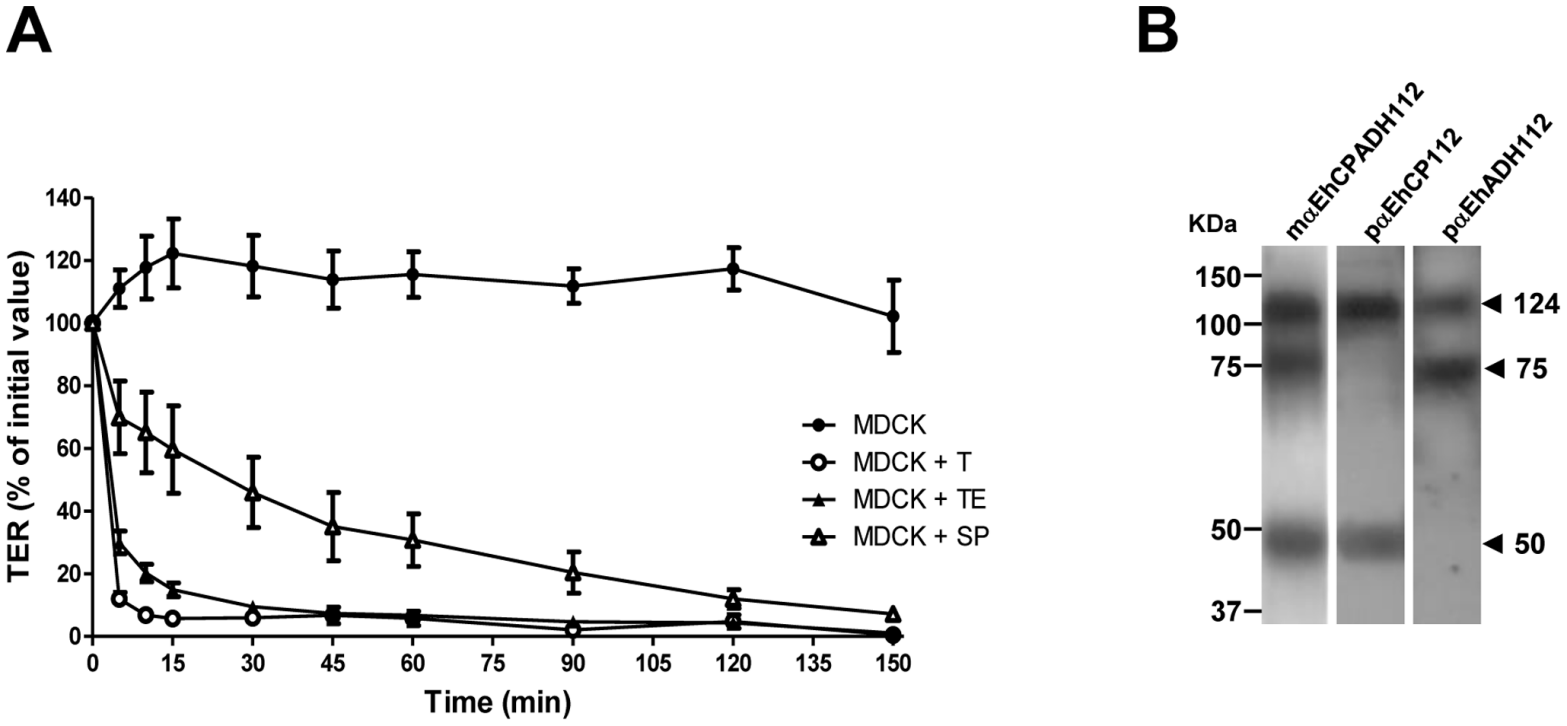
*Entamoeba histolytica* induces epithelial barrier disruption. **A)** MDCK monolayers were incubated with T, TE or SP for 2.5 h and TER was evaluated as described in Materials and Methods at indicated times. TER was normalized according to the initial value for each transwell (∼2,220 Ω·cm^2^). Means and standard errors are represented for each time point. **B)** Trophozoites were lysed, separated by SDS-PAGE and analysed by Western blot with the indicated antibodies. Left: molecular weight standards. Arrowheads: molecular weights of immunodetected proteins.

**Figure 2:**
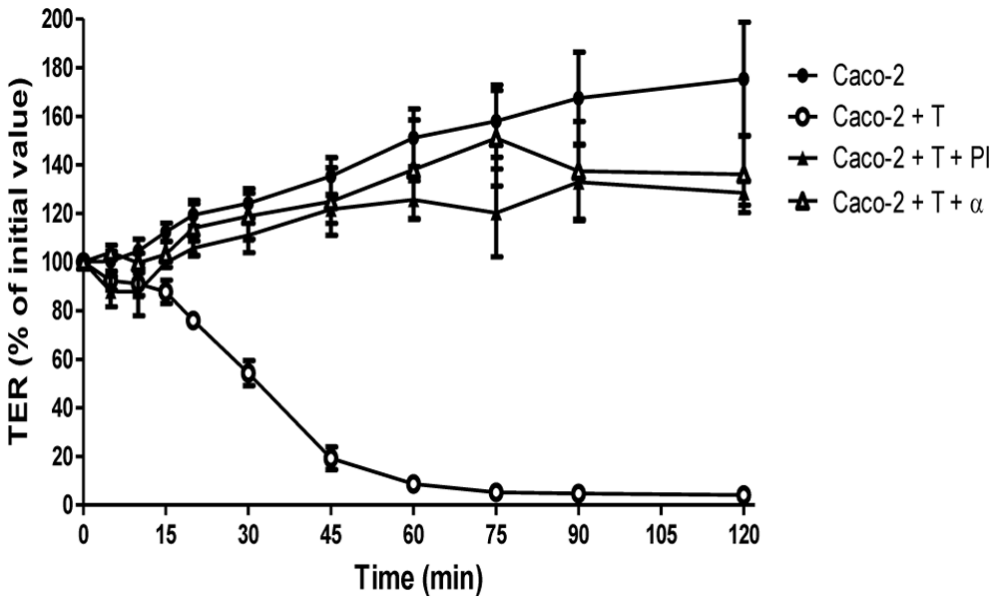
EhCPADH112 induces epithelial barrier disruption in Caco-2 cells. **A)** Caco-2 cells were incubated with T or T pre-incubated with protease inhibitors (PI) or mαEhCPADH112 antibody (α). TER was normalized according to the initial value for each transwell (∼600 Ω·cm^2^). Means and standard errors are represented for each time point.

Incubation of MDCK monolayers with either *E. histolytica* trophozoites (T), trophozoite extracts (TE) or secreted products (SP) induce progressive disruption of the paracellular barrier as measured by TER (Fig. 1A). We also performed a dose-response curve for the trophozoites and found that the extent of barrier disruption depends on the number of trophozoites added to the monolayer, excluding a general toxic effect of the trophozoites for the MDCK monolayer (Figure S1A). Since *E. histolytica* infects the human intestinal tract, we examined if the same effect occurred in the human colonic epithelial cell line Caco-2. Indeed, we found a similar dose-dependent response for barrier disruption (Figure S1B). More importantly, when treating Caco-2 monolayers with TE in the presence of a protease inhibitor cocktail or a monoclonal blocking antibody against the EhCPADH112 complex, we observed an almost complete inhibition of the TER drop proving a specific role for this complex in barrier disruption (Figure 2).

To prove that TE indeed contain the proteins of the EhCPADH112 complex, we performed Western blots using antibodies against the whole complex and against its single components (EhCP112 and EhCPADH112). As shown in Figure 1B, our antibodies recognized the 124 kDa band of the whole complex (mαEhCPADH112), the 50 kDa band of the cysteine protease (pαEhCP112) and the 75 kDa band of the adhesin (pαEhADH112) as expected [5], [11], [16].

### The *E. histolytica* EhCPADH112 Complex Localizes at TJs

**Figure 3:**
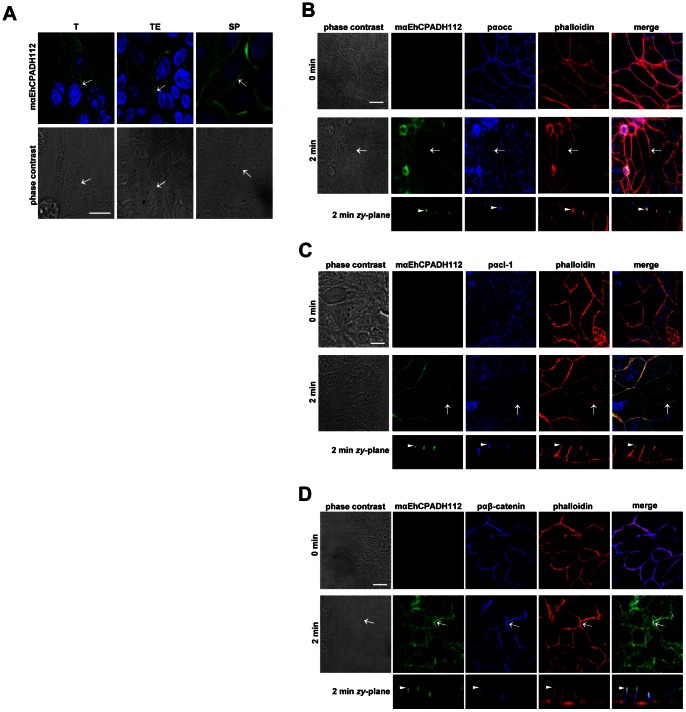
The EhCPADH112 complex localizes at TJ of MDCK monolayers. **A)** MDCK cells were incubated with T, TE or SP for 2 min. Upper panel: the EhCPADH112 complex was identified by mαEhCPADH112 antibody and nuclei were stained with DAPI. Lower panel: phase contrast images of MDCK cells. **B-D)** MDCK cells untreated (0 min) and incubated with TE for 2 min were stained with specific antibodies against EhCPADH112 (green) and occludin (blue) (**B**), claudin-1 (blue) (**C**) or β-catenin (blue) (**D**). Actin was stained with TRITC-phalloidin (red). Arrows: protein localization at cell borders. *Zy*-planes: co-localization at TJs (arrowheads). Bars = 10 µm.

To gain a better understanding of the role of the EhCPADH112 complex in barrier disruption, we performed IF stainings of MDCK cell monolayers that were in contact with trophozoites. Confocal microscopy images showed that after contact with T, TE or SP for 2 min, the EhCPADH112 complex can be detected at epithelial cell borders (Fig. 3A, arrows). Furthermore, we performed co-stainings using antibodies against the EhCPADH112 complex together with either the TJ markers occludin and claudin-1 or the adherence junction (AJ) marker β-catenin and phalloidin to label actin filaments. MDCK monolayers showed the typical honeycomb pattern for occludin, claudin-1, β-catenin and actin that remains intact after short interaction (2 min.) of epithelial cells with TE (Fig. 3B, 3C and 3D). As shown in figures 3B and 3C (merge panels), we observed co-localization of the EhCPADH112 complex with occludin, claudin-1 and actin at cell borders. However, EhCPADH112 co-localize with occludin and claudin-1 in the most apical portion of the lateral membrane where TJs are located (Fig. 3B and 3C, *zy*-planes, arrowheads). On the other hand, EhCPADH112 co-localization with β-catenin and actin was only partial in the apical junctional complex in regions corresponding to TJ rather than AJ (Figure 3D).

### EhCP112 and EhADH112 Proteins also Localize at TJs

**Figure 4:**
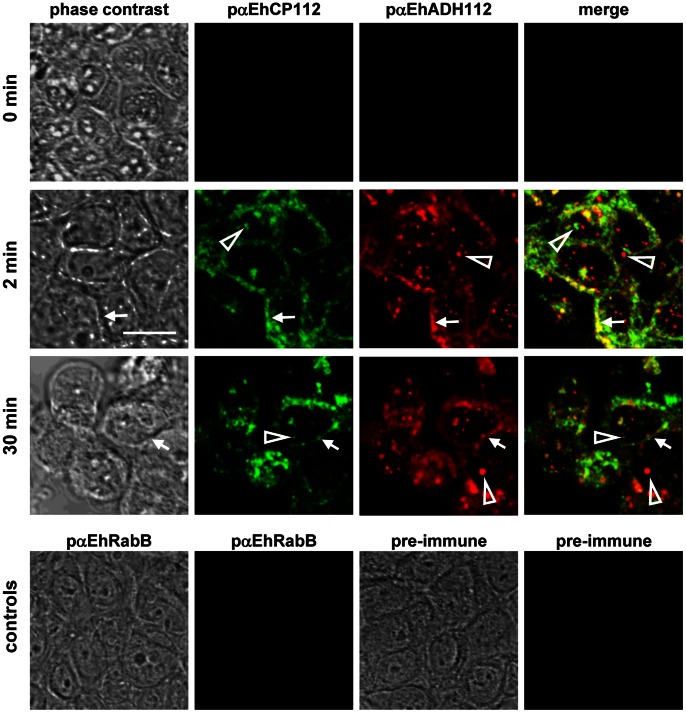
EhCP112 and EhADH112 co-localize at cellular borders of MDCK monolayers. MDCK monolayers were left untreated (0 min) or incubated with TE for 2 or 30 min. EhCP112 (green) and EhADH112 (red) were labeled with specific antibodies. Arrows: EhCP112 and EhADH112 co-localization at cell borders. Empty arrowheads: separate localization of EhCP112 or EhADH112 in vesicles. Controls: Untreated MDCK cells did not show any signals when using pαEhCP112, pαEhADH112, pαEhRabB or pre-immune serum. Bar = 10 µm.

We separately studied the interaction of both proteins forming the EhCPADH112 complex, EhCP112 and EhADH112, with MDCK epithelial cells after incubation with TE (Figure 4). At early times of interaction (2 min), phase contrast images showed no visible damage of MDCK cells, whereas at 30 min, the majority of epithelial cells were rounded, detached from the substrate and morphologically damaged. After 2 min incubation, EhCP112 protein was located at MDCK cell borders and in vesicle-like structures inside the cells (Fig. 4, 2 min, green panel, arrows and empty arrowheads, respectively). At 30 min, EhCP112 fluorescence was diffuse at cell borders (Fig. 4, 30 min, green panel, arrows) and positive vesicles were still detected (Fig. 4, 30 min, green panel, empty arrowheads). The EhADH112 protein displayed a similar distribution pattern than that obtained for EhCP112 (Fig. 4, red panel). However, pαEhADH112 antibody detected a larger number of vesicles than pαEhCP112. Merged images revealed a co-localization of EhCP112 and EhADH112 proteins at epithelial cell borders (Fig. 4, merge panels, arrows) and in some intracellular vesicles. However, some EhCP112 and EhADH112 molecules were found separately (Fig. 4, merge images, empty arrowheads).

On the other hand, in MDCK cells that were not in contact with TE, the antibodies against EhCP112, EhADH112 or the *E. histolytica* EhRabB, a cytoplasmic protein used as control, as well as a pre-immune serum showed no signals (Fig. 4, controls).We also detected co-localization of both EhCP112 and EhADH112 with occludin (Figures S2A and B). After 2 min interaction with TE, The pαEhCP112 antibody gave a similar pattern to the one described in figure 1 (Fig. S2A, 2 min, green panel). Merged images and z*y*-sections revealed co-localization of occludin and EhCP112 at TJs. Nevertheless, in some experiments, EhCP112 was also detected along the lateral membrane (Fig. S2A, *zy*-planes), similar to EhCPADH112 at 30 min interaction (Fig. S3, 30 min, red panel, arrowheads). These results suggest that EhCP112 penetrates the paracellular pathway farther than TJs. When cellular damage was visible at 30 min (Fig. S2A, 30 min, phases contrast panel), co-localization of EhCP112 and occludin could be detected at intercellular junctions (Fig. S2A, merge panel, 30 min, arrows). and more prominently in vesicles within the cells (Fig. S2A, 30 min, merge panel, empty arrowheads). The pαEhADH112 antibody gave a similar fluorescence patterns, although the honeycomb pattern appeared more defined even after 30 min interaction (Fig. S2B).

Our findings suggest that after early contact of trophozoites with epithelial cells, EhCP112 and EhADH112 can be detected in co-localization with TJ markers and inside cells. However, the interaction of EhCPADH112 with TJ proteins does not seem to have a detrimental effect on the cytoskeleton since we barely observed disruption of actin filaments after short contact with TE (figures 3B–D) or after 30 min exposure (Figure S3, 30 min, red panel).

### EhCP112 and EhADH112 Proteins Associate with TJ Components

**Figure 5:**
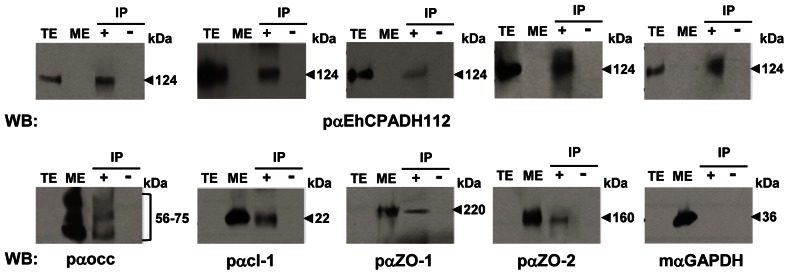
The EhCPADH112 complex co-immunoprecipitates with TJ components. EhCPADH112 was immunoprecipitated from MDCK cell lysates incubated with TE for 30 min, and analysed by Western blot with the indicated antibodies. IP, lane +: immunoprecipitation using pαEhCPADH112 antibody. IP, lane –: immunoprecipitation using pre-immune serum as control. Lane TE: whole trophozoite extracts only. Lane ME: whole MDCK cell extracts only. Upper panel: immunodetection using pαEHCPADH112 antibody. Lower panel: immunodetection with antibodies against TJ proteins or against GAPDH. Arrowheads: molecular weight of immunodetected proteins.

To confirm the interaction of EhCP112 and EhADH112 with occludin and other TJ proteins, we performed immunoprecipitation experiments using the pαEhCPADH112 antibody. In the immunoprecipitates we detected the expected 124 kDa band corresponding to the EhCPADH112 complex (Fig. 5, upper panel, lanes “+”), co-migrating with the same band in TE (Fig. 5, upper panel, lanes TE). The pαEhCPADH112 antibody did not recognize any band in MDCK cell extracts or in immunoprecipitates obtained with pre-immune serum (Fig. 5, upper panel, lanes ME and “−”, respectively). Then, the same membranes were washed and re-probed with specific polyclonal antibodies against occludin and other TJ components (claudin-1, ZO-1 and ZO-2). Using the pαoccludin antibody, we detected a band triplet in the range of 56–75 kDa corresponding to the original reported band for occludin of 65 kDa [36] and differently phosphorylated [37] or alternatively spliced isoforms [38] (Fig. 5, lower panel, lane “+”) in immunoprecipitates and in MDCK whole cell extracts (Fig. 5, lower panel, lane ME). Additionally, in EhCPADH112 immunoprecipitates, pαcl-1 detected a 22 kDa band, whereas pαZO-1 and pαZO-2 revealed 220 and 160 kDa bands, respectively. All proteins co-migrated with their respective ones in MDCK whole cell extracts (Fig. 5, lower panel, lanes “+” and ME, respectively). Antibodies against TJ components did not recognize any protein in TE (Fig. 5, lower panel, lanes TE). As another negative control, an antibody against GAPDH did not detect bands in EhCPADH112 immunoprecipitates (Fig. 5, lower panel, mαGAPDH). Immunoprecipitates using pre-immune serum showed no cross-reactivity (Fig. 5, lanes “−”). These results demonstrate that occludin, claudin-1, ZO-1 and ZO-2 interact with the EhCPADH112 protein complex.

### EhCP112 and EhADH112 Interact Preferably with Claudin-1 and Occludin

**Figure 6:**
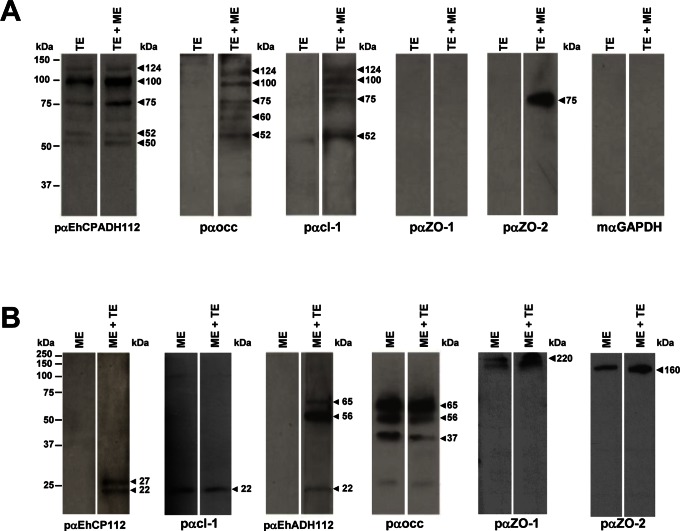
EhCP112 and EhADH112 associate to occludin and claudin-1. **A)** TE were separated by SDS-PAGE, transferred onto nitrocellulose membranes and incubated with ME as described in Materials and Methods. Membranes were then probed with antibodies against EhCPADH112, occludin, claudin-1, ZO-1, ZO-2 and GAPDH as indicated to detect interactions among the respective proteins. Lane TE: trophozoite extracts only. Lane TE+ME: trophozoite extracts incubated with MDCK extracts. **B)** MDCK extracts were separated by SDS-PAGE, transferred onto nitrocellulose membranes and incubated with TE. Subsequently, membranes were probed with antibodies against EhCP112, claudin-1, EhADH112, occludin, ZO-1 and ZO-2 as indicated. Lane ME: MDCK extracts only. Lane ME+TE: MDCK cell extracts incubated with trophozoite extracts. Left: molecular weight standards. Arrowheads: molecular weights of detected proteins.

To investigate a specific interaction of EhCP112 and EhADH112 with each of the above mentioned TJ proteins, we performed overlay experiments. In contrast to mαEhCPADH112 (compare Figure 1B), pαEhCPADH112 recognized *E. histolytica* proteins of 124, 100, 75, 52 and 50 kDa (Fig. 6A, first panel, arrowheads). The 124 kDa band corresponds to the EhCPADH112 complex [5], the 100 kDa band represents the EhADH112-like proteins [15], the 75 kDa band corresponds to EhADH112 and the 50/52 kDa bands represent EhCP112 forms [5]. In membranes containing TE that were later incubated with MDCK lysates (ME), the pαocc antibody detected the presence of occludin associated to parasite proteins of 124, 100, 75, 60 and 52 kDa (Fig. 6A, second panel, lane TE+ME). Meanwhile, pαcl-1 revealed claudin-1 bound to *E. histolytica* proteins of 124, 100, 75 and 52 kDa (Fig. 6A, third panel, lane TE+ME); pαZO-1 showed no interaction of ZO-1 with any of the mentioned protein bands (Fig. 6A, fourth panel); and pαZO-2 exhibited a strong interaction of ZO-2 with the 75 kDa EhADH112 protein (Fig. 6A, fifth panel, lane TE+ME). As controls, pαocc, pαcl-1, pαZO-1 and pαZO-2 antibodies did not react with trophozoite proteins that were not in contact with MDCK extracts (Fig. 6A, lanes TE). As an additional control, we used mαGAPDH that did not detect any trophozoite proteins (Fig. 6A, last panel). These findings suggest that occludin and claudin-1 show affinity for the whole EhCPADH112 complex, whereas ZO-2 only interacts with EhADH112.

Reverse overlay experiments were performed using MDCK cell proteins immobilized on membranes and incubated with TE. Notably, in these membranes the pαEhCP112 antibody revealed a doublet of 22 and 27 kDa (Fig. 6B, first panel, lane ME+TE). The 22 kDa protein co-migrated with claudin-1 in ME (Fig. 6B, second panel, lane ME+TE). By contrast, pαEhADH112 bound to 22, 56 and 60 kDa MDCK proteins (Fig. 6B, third panel, lane ME+TE), corresponding to bands reported for claudin-1 (22 kDa) and occludin (56 and 60 kDa; Fig. 5B, second and fourth panel, lane ME+TE). However, in these experiments bands for the molecular weight expected for ZO-1 or ZO-2 bound to EhCP112 or EhADH112 did not appear (Fig. 6B, compare first and third with fifth and sixth panels). As controls, pαEhCP112 and pαEhADH112 antibodies did not detect signals in MDCK cell lysates (Fig. 6B, first and third panels, lanes ME). This data shows that EhCP112 and EhADH112 interact preferably with occludin and claudin-1.

### Trophozoite Extracts Degrade TJ Components

**Figure 7:**
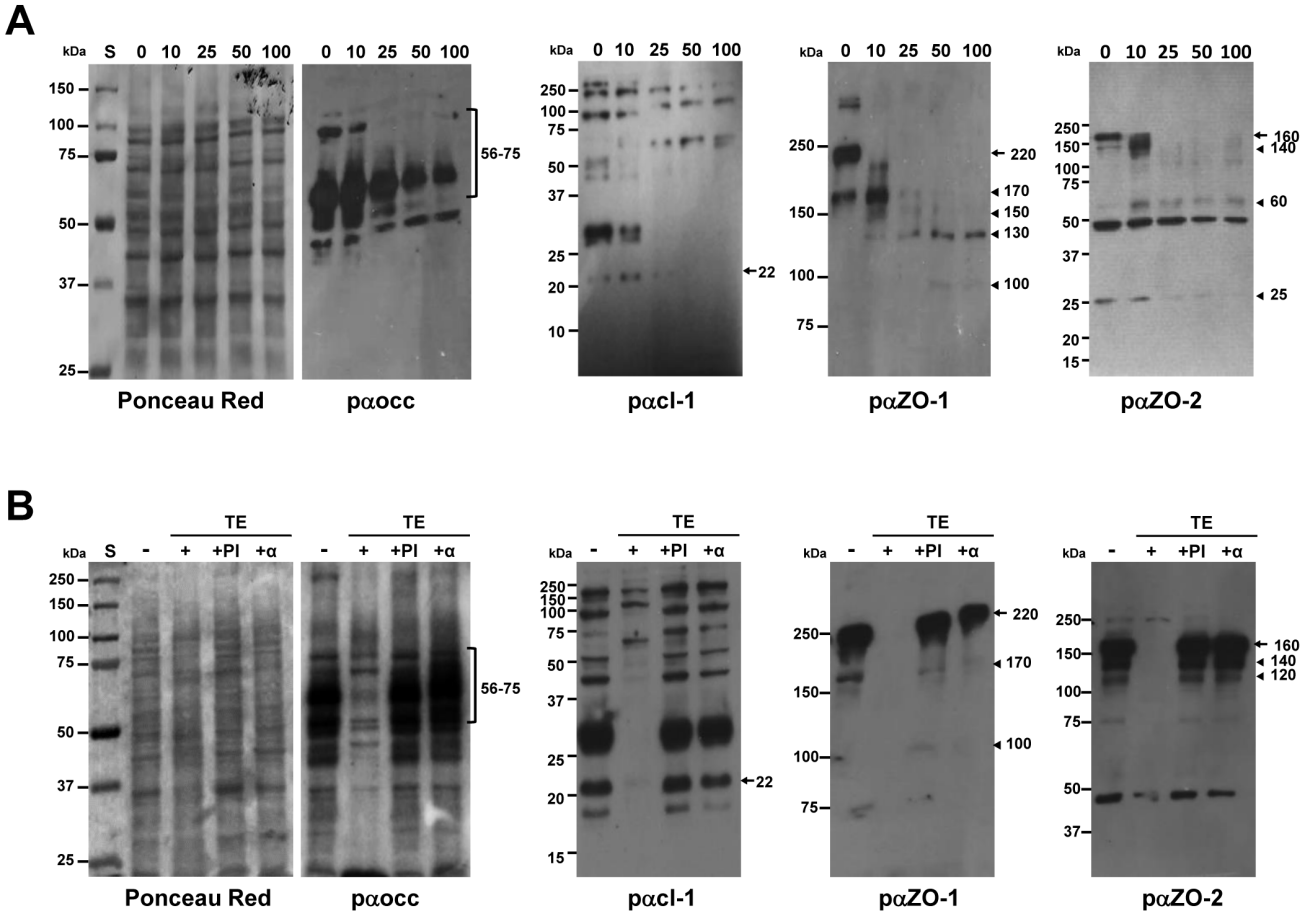
TJ components are degraded in a dose-dependent manner by TE. **A)** MDCK cells were left untreated (0) or incubated for 30 min with TE from 10, 25, 50 or 100×10^3^ trophozoites as described in Materials and Methods. Cells were then lysed and processed for Western blot analysis using the indicated antibodies. **B)** MDCK cells were left untreated (-), treated with TE (from 100×10^3^ trophozoites) alone (+), treated with TE pre-incubated with protease inhibitors (+PI) or with TE pre-incubated with the mαEhCPADH112 antibody (+α). All MDCK cells were lysed, separated by SDS-PAGE and analysed by Western blot using antibodies against occludin, claudin-1, ZO-1 and ZO-2 as indicated. The first panels in A and B show Ponceau Red stainings of the membrane used to detect occludin. Left: molecular weight standards (S). Arrows: molecular weights of TJ proteins. Arrowheads: degradation products.

To evaluate any proteolytic effect of TE on TJs as a mechanism of barrier disruption, we incubated MDCK cells for 30 min with different concentrations of TE. Ponceau Red stainings of the membranes showed similar protein patterns for untreated MDCK cells and those incubated with TE (Fig. 7A and 7B, first panels, Ponceau Red). Western blot assays of MDCK cell lysates that were not in contact with TE showed the expected band triplet for occludin (Fig. 7A, second panel, 0 min, arrows) [36], [37], [38], that is diminished in a dose-dependent manner when incubated with different amounts of TE. (Fig. 7A, second panel, TE 10–100, arrows). Likewise, control MDCK cells revealed the expected band of 22 kDa for claudin-1 (Fig. 7, third panel, 0 min, arrow) that disappeared after treatment with TE (Fig. 7, third panel, TE 25–100, arrow). In both treated and untreated cells, additional bands of higher molecular weight were present that have been interpreted by others as claudin oligomers [39](Fig. 7, third panel). In MDCK cells that were not incubated with TE, we identified a 220 kDa band, using pαZO-1 [40] (Fig. 7, fourth panel, 0 min, arrow) that disappeared when epithelial cells were incubated with different amounts of TE (Fig. 7A, fourth panel, 10–100, arrow). Instead, bands of 170, 150, 130 and 100 kDa appeared likely presenting degradation products (Fig. 7A, fourth panel, 10–100, arrowheads). In untreated MDCK cells, pαZO-2 revealed a 160 kDa protein as expected [41] (Fig. 7A, fifth panel, pαZO-2, C, arrow) that also disappeared when cells were treated with different concentrations of TE (Fig. 7A, fifth panel, 10–100, arrows). Additionally new bands of 140, 60 and 25 kDa showed up, probably corresponding to degradation products (Fig. 7A, fifth panel, 10–100, arrowheads). These data imply that occludin, claudin-1, ZO-1 and ZO-2 are degraded by proteases present in TE.

To corroborate this notion we performed degradation experiments with TE (100×10^3^ trophozoites) for 30 min alone or in the presence of either a protease inhibitor cocktail or the monoclonal antibody against the EhCPADH112 complex (Figure 7B). Strikingly, both the protease inhibitor cocktail (+PI) and the antibody (+α) completely reversed the degradation of occludin, claudin-1, ZO-1 and ZO-2, indicating that the proteolytic activity of the EhCPADH112 complex is responsible for the degradation of TJ proteins after *E. histolytica* treatment.

### Trophozoite Extracts Induce Apoptosis and Autophagy but not Necrosis in MDCK Cells

**Figure 8:**
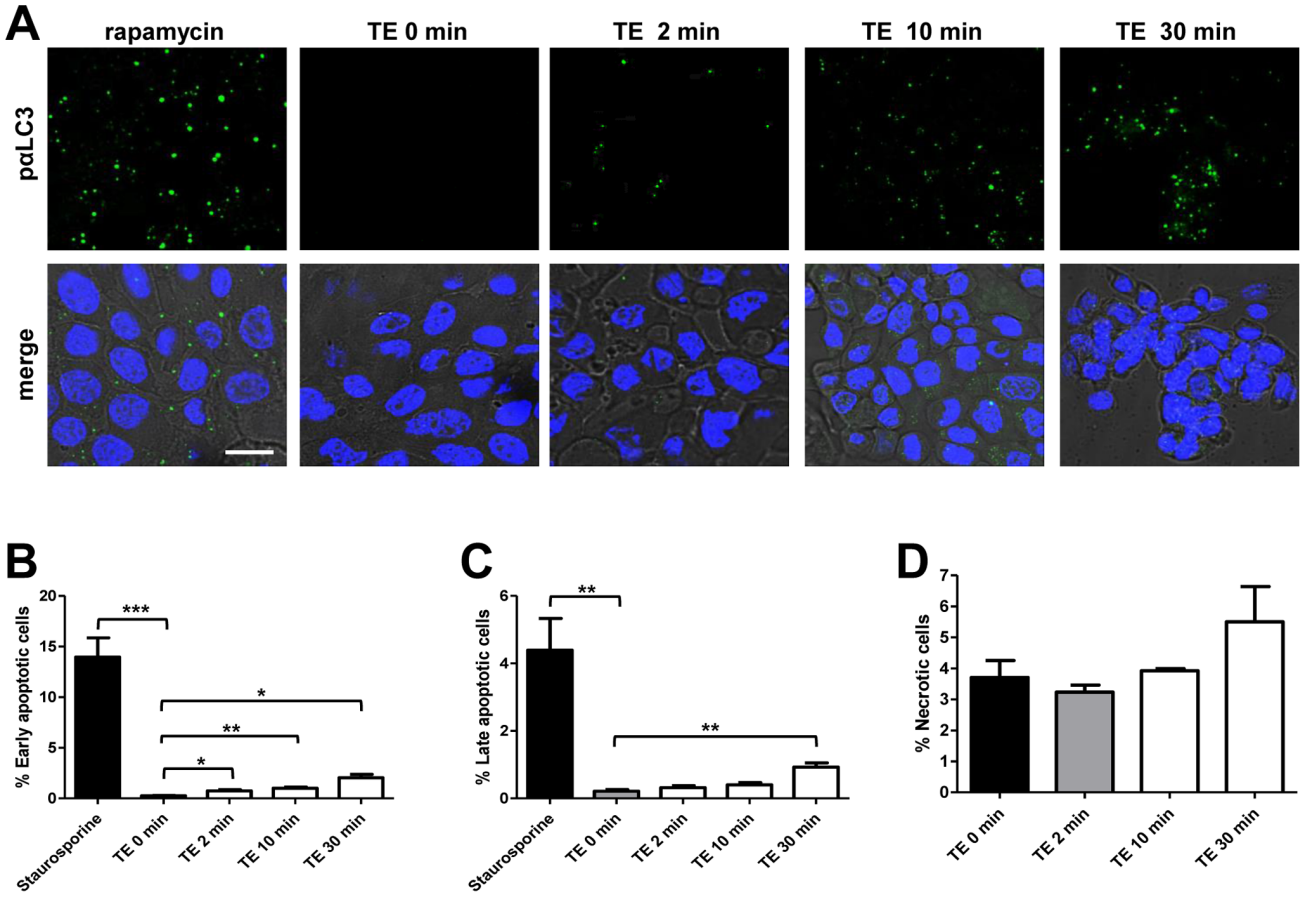
*Entamoeba histolytica* extracts induce autophagy and apoptosis but not necrosis when interacting with MDKC cells. MDCK cells were left untreated (0 min) or treated with TE for 2, 10 and 30 min. **A)** Epithelial cells were immunofluorescently labelled with an antibody against LC3 (pαLC3 antibody, green) to determine the formation of autophagosomal vacuoles. As positive control, MDCK cells were incubated with rapamycin. Nuclei were stained with DAPI. Bar = 10 µm. **B–C)** MDCK cells were examined for early (**B**) and late (**C**) apoptosis by flow cytometric analysis of annexin V and propidium iodide. As positive control, MDCK cells were treated with staurosporine. **D)** To detect necrosis, positive cells for propidium iodide staining were evaluated by flow cytometric analysis. Graphs in **B–D** represent four independent experiments and data are displayed as mean ± standard error of the mean. *P<0.05, **P<0.01, ***P<0.001.

To investigate if an increased incidence of cell death could contribute to barrier disruption in epithelial cells after contact with TE, we performed assays to determine apoptosis, autophagy and necrosis. To determine if TE-treated MDCK cells would undergo autophagy, we performed immunofluorescence stainings for LC3, a protein involved in the formation of autophagosomes. TE treatment indeed induced the formation of autophagosomal vacuoles over time (Fig. 8A, upper panel). Only a few cells were positive after 2 min of treatment (Fig. 8A, second panel), whereas several cells were positive for LC3 after 10 min and 30 min of treatment (Fig. 8A, third and four panels, respectively). As positive control, MDCK cells were incubated with rapamycin (Fig. 8A, first panel).

For apoptosis and necrosis determination, we used combined annexin V and propidium iodide stainings followed by flow cytometry analysis. The results revealed that TE significantly induced an increase in early apoptotic MDCK cells as quickly as 2 min post-treatment (Figure 8B). The incidence of early apoptotic MDCK cells (positive only for annexin V) was even higher after 10 and 30 min post-treatment. By contrast, the amount of late apoptotic MDCK cells (positive for both annexin V and propidium iodide) was only significantly increased after 30 min of treatment with TE (Figure 8B). Staurosporine-treated MDCK cells were used as positive control. Importantly, we never detected a significant increase of necrotic cells in MDCK cells treated with TE (positive only for propidium iodide), in comparison with untreated cells, (Fig. 8D).

Our data suggest that TE may contribute to epithelial barrier disruption via mechanisms that provoke cell death by autophagy and apoptosis, but not necrosis.

## Discussion

Interaction between *E. histolytica* trophozoites and the host intestine initially requires attachment of the parasite to the epithelium to allow subsequent invasion. The first evident morphological modification of epithelial cells after contact with trophozoites is a widening of intercellular spaces due to gradual separation of adjacent cells that disturbs the epithelial barrier [12], [24]. A drop of TER is an early signal of epithelial disruption, as it has been shown here and by others [12], [25], [26], [27]. However, the molecular mechanisms by which trophozoites alter the intestinal barrier remain poorly understood.

Among the parasite molecules that could participate in this process is the EhCPADH112 complex, which is mainly located at the plasma membrane of trophozoites [5]. Remarkably, EhCPADH112 is secreted as a complex and its components (EhCP112 and EhADH112) have also been detected in the extracellular milieu [11].

To investigate if this complex contributes to epithelial damage, we used trophozoites pre-incubated with a specific antibody against EhCPADH112 and observed that the TER drop is prevented. Similar observations were obtained when trophozoites were pre-incubated with protease inhibitors suggesting that the protease activity of EhCP112 is also involved in provoking barrier disruption.

To gain better understanding of the mechanisms whereby EhCPADH112 causes barrier disruption, we studied the localization of this complex in epithelial cells treated with trophozoite extracts. First, we proved by confocal microscopy experiments, that after parasite-MDCK cell interaction, the EhCPADH112 complex and its components, are indeed found at TJs of MDCK epithelial cells in co-localization with occludin and claudin-1. At early interaction times, the EhCPADH112 complex is localized only at TJ, but not along the whole lateral membrane. Interestingly, in some cases, EhCP112 was located along the lateral membrane suggesting that this protease might penetrate beyond TJs possibly interacting also with AJs or desmosomes. This can be explained by the proteolytic activity of EhCP112 that destroys molecules and even entire cells [11] thus facilitating tissue invasion by the parasite. Location at epithelial cellular borders was specific for EhCPADH112, EhCP112 and EhADH112, since the cytoplasmic parasite protein EhRabB, did not attach to MDCK cells. In our assays, we also found both EhCPADH112and its single components inside cells, suggesting that EhCP112 and EhADH112 alone or as a complex contribute to target cell destruction. Many questions arise from these findings, but a working hypothesis may include the possibility that after contact with target molecules, the EhCPADH112 complex suffers conformational changes that allow its disassembly and enable its single components to interact with different host molecules such as epithelial cell membrane proteins.

Additionally, it has long been known that disruption of the actin cytoskeleton causes extensive tight junction disruption [42]. Many pathophysiological effectors, including proinflammatory cytokines and infectious agents, induce the contraction of actin filaments docked to TJs, thus contributing to TJ opening [43]. However, our findings clearly show that the actin cytoskeleton is not involved in the TER drop, since its cortical localization is barely affected even at late times of cell-parasite interaction.

According to other authors, morphological changes observed in epithelia after interaction with trophozoites are accompanied by a rapid decrease of TER and an increased paracellular permeability of epithelial cell layers [12], [26], [44] strongly suggesting a selective disturbance of TJ structures by the parasite. In colonic enteric cells incubated with trophozoites, the 170 kDa subunit of the Gal/GalNAc-specific lectin and the lipophosphopeptidoglycan (LPPG) are transferred from the parasite to the enteric cell layer and concentrated at cell-cell borders [45], [46]. However, double immunostaining assays using antibodies against molecules of the TJ structure and against both the parasite lectin and LPPG demonstrated that TJs are localized apically in relation to the transferred lectin and LPPG signals [45], [46] suggesting that TJ proteins are not the binding molecules for lectin and LPPG. In fact, the lectin signal merges with the staining pattern of E-cadherin, an epithelial integral protein of AJs located basally to TJs [45]. Another parasite protein that had been involved in the opening of TJs is PGE_2_, although a direct association of PGE_2_ with TJ components has not been demonstrated yet. Instead, PGE_2_ uses a selective signalling transduced via the EP receptor which is probably located at the apical membrane of epithelial cells [27]. These results demonstrate the complexity of molecular events and the great number of molecules participating in early host-parasite relationship. As epithelial damage progresses, EhCPADH112 moves further to the paracellular space and distributes along the lateral membrane whileEhCP112, EhADH112 and occludin co-localize mainly inside cells, possibly due to cell membrane rupture or an active internalization.

Immunoprecipitation and overlay assays strongly suggest that the EhCPADH112 complex preferentially associates with occludin and claudin-1. In contrast to our immunoprecipitation assays, overlay experiments did not detect any association between EhCPADH112 or its derivatives with ZO-1. Further experimental approaches are needed to clarify this, since TJ proteins were denaturated in overlay assays, whereas native proteins had been used for immunoprecipitation experiments. Nevertheless, our results also suggest that during or after target cell-parasite interaction, molecules forming the EhCPADH112 complex could interact separately with TJ proteins provoking disruption of epithelial monolayers. Accordingly, EhCP112 seems to mainly interact with claudin-1, whereas EhADH112 exhibits affinity to both occludin and claudin-1. Thus, our findings revealed a novel interaction between parasite proteins and several TJ molecules. Since EhCP112 belongs to the papain family of proteases [18], we expected that EhCPADH112 or EhCP112 interaction with other proteins could result in their degradation. This hypothesis was proven by the analysis of TJ protein integrity after contact with TE. Of note, trophozoite extracts affected TJ proteins in a dose-dependent manner. Occludin was slightly degraded with the uppermost band of the triplet being the most affected. As it has been reported, highly phosphorylated forms of occludin are concentrated at TJs [38], thus our findings suggest that TE treatment could be mainly affecting functional occludin forms. In addition, the monomeric form of claudin-1 was also diminished by TE, although other oligomeric forms were only slightly degraded. Interestingly, the adaptor proteins ZO-1 and ZO-2 were highly digested, even though they are located inside cells and are not immediately exposed to EhCP112 or EhCPADH112. However, the cytoplasmic regions of occludin and claudin-1 interact with scaffold proteins such as ZOs [20]. Thus, an interaction of EhCPADH112 with integral proteins could affect their affinity for ZOs leading to dissociation and proteasomal degradation.

Our results suggest that TJ integral proteins are more resistant to proteolytic degradation than adaptor proteins, or that integral proteins are protected by an unknown mechanism. The specific participation of the EhCPADH112 complex in differential degradation of TJ components could be proven using trophozoites pre-incubated with the mαEhCPADH112. Furthermore, when we used trophozoites incubated with protease inhibitors, degradation of TJ components was also inhibited strengthening the hypothesis that the proteolytic activity of EhCP112 plays an important role in mediating epithelial damage. However, degradation cannot be only attributed to EhCP112 since *E. histolytica* expresses many other proteases that are secreted by trophozoites during invasion. For example, the cysteine protease 5 (EhCP5) is secreted by trophozoites and binds to its epithelial receptor, α_ν_β_3_ integrin [47]. Nevertheless, it is important to note that according to our previous *in silico* analysis and western blot assays, the pαEhCP112 antibody used here does not recognize other *E. histolytica* cysteine proteases (data not shown).

Other research groups also found degradation of ZO-1 and ZO-2 in colonic cells, although they did not observe occludin or claudin-1 degradation [26], [27], [44]. These results could be explained because degradation of occludin and claudin-1 was masked under those experimental conditions or perhaps it was not resolved under the SDS-PAGE parameters used in their studies. Leroy et al (2000a) found that during contact of trophozoites with cell monolayers, ZO-2 is dephosphorylated and separated from ZO-1. Furthermore, Lejeune, et al (2011) observed an increase in claudin-4 expression and internalization of this protein during trophozoite-epithelial cell interactions. These findings confirm that the most affected TJ proteins are ZO-1 and ZO-2, whereas occludin and claudin-1 are degraded to a lesser extent. We could also show early epithelial barrier disruption in the human colonic epithelial cell line Caco-2 that was specifically mediated by the proteolytic activity of the EhCPADH112 complex. However, it needs to be determined in future studies if the degradation observed in MDCK cells also accounts for barrier disruption in Caco-2 cells.

Partial protection of occludin and claudin-1 from degradation in our experiments could be due to their association with EhADH112 that could mask the cleavage site for EhCP112. Thus, it is tempting to speculate that EhCPADH112/occludin or EhCPADH112/claudin-1 interactions are stronger than occludin- or claudin-1 homodimers (Fig. 8). This mechanism could be also an explanation for the TER drop [12], [26],. Similar mechanisms have been described for other pathogens such as *Clostridium perfringens*, reovirus and coxsackievirus, that use TJ integral proteins (claudins, JAM-A and CAR, respectively) as receptors to uncouple the paracellular barrier and penetrate deeper into tissues [43].

**Figure 9:**
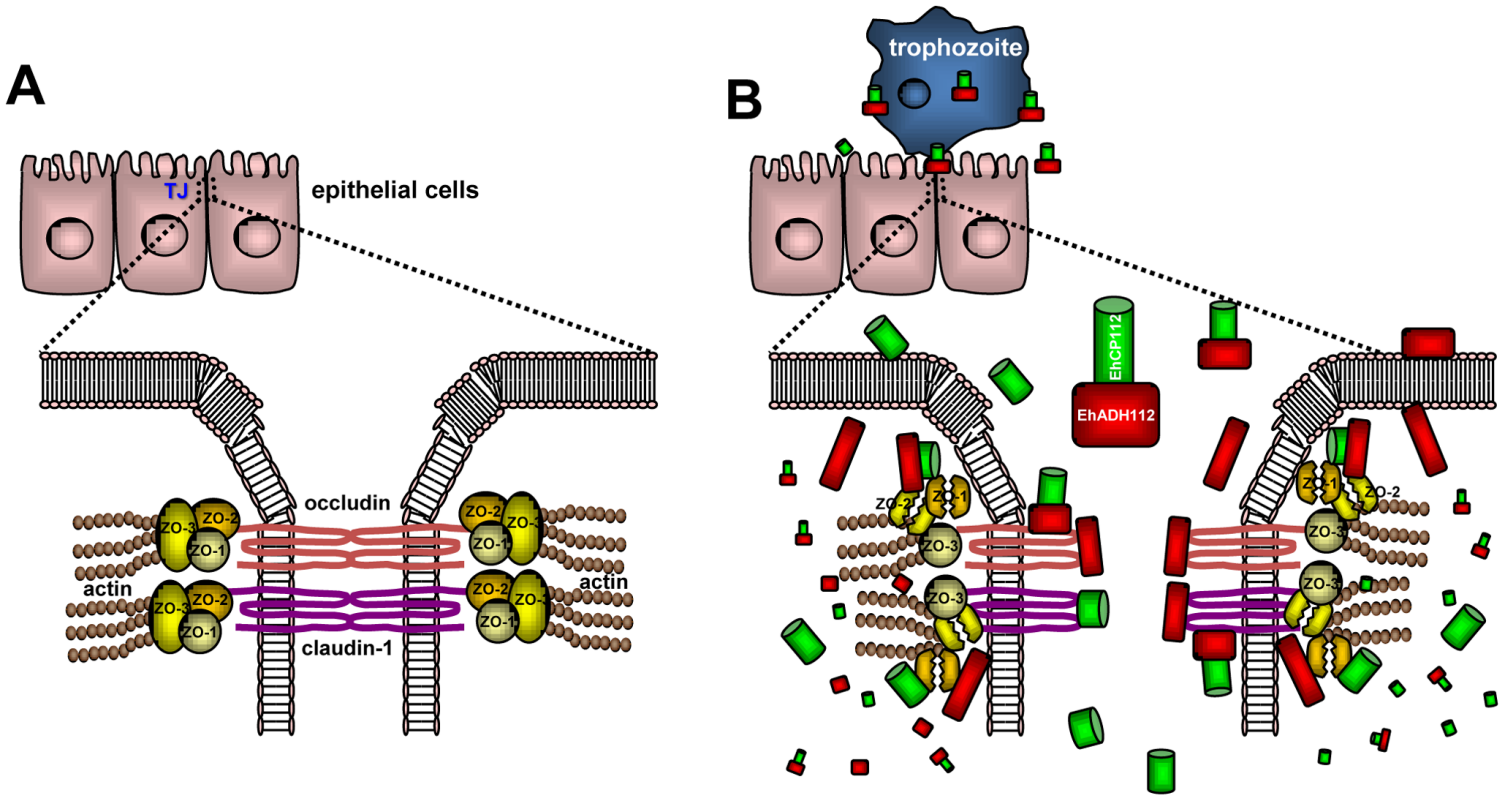
Working model explaining TJ disruption by the EhCPADH112 complex. **A)** TJs are formed at the border between the apical and the basolateral sides of neighbouring cells. Among their integral components are occludin and claudin-1 that interact with the actin cytoskeleton via members of the ZO family of proteins. **B)** Trophozoites reach the intercellular region of epithelial cells exposing the EhCPADH112 complex (red rectangles and green cylinders) at their plasma membrane or secreting it into the media. Alternatively, the EhCPADH112 complex could undergo conformational changes resulting in the separation of EhCP112 (green cylinders) from EhADH112 (red rectangles). Next, EhCPADH112, EhCP112 and EhADH112 bind to occludin and claudin-1 causing disassembly of epithelial cell-cell interactions and a drop in TER. Once inside epithelial cells, *Entamoeba* proteases such as EhCP112 can degrade intracellular TJ proteins such as ZO-1 and ZO-2. As a consequence of TJ disruption, other parasite proteins may enter through the paracellular pathway to trigger epithelial damage.

EhCP112 and EhADH112 are known to be expressed at the trophozoite plasma membrane [5], [16], but they can also be released [11] to reach TJs. Then, together with other *E. histolytica* molecules, EhCPADH112 interacts with target cell proteins, including TJ adaptor molecules, to degrade them and cause permanent tissue damage (Fig. 9). When TJs are disturbed, Gal/GalNAc lectin, LPPG, PGE_2_, EhCP5 and other molecules [6], [27], [45], [47] could arrive at the basolateral membranes reaching their receptors or degrading membrane proteins to trigger cell damage (Fig. 9). In this regard, while this paper was in revision, another paper has been published showing that EhCP-A5 indeed has a proinflammatory and barrier disrupting effect on the intestinal epithelial barrier [48]. Interestingly, they performed experiments in Mucin2-KO mice and showed that mucin-2 has a protective effect against *E. histolytica* infections and particularly, against the epithelial damage elicited by EhCP-A5. Whether mucins also protect against epithelial damage mediated by EhCPADH112 needs to be determined in future studies.

During infection, trophozoites of *E. histolytica* can lyse host cells. So far, the only signalling mechanism known responsible for amoeba-induced host cell death is induction of apoptosis. The authors showed that this induction is on the one hand caspase-dependent but also dependent on NOX1-derived ROS [49], [50], [51]. However, it remains unknown if apoptosis also contributes to epithelial barrier disruption by *E. histolytica*. Our findings show that at early times of interaction, *E. histolytica* slightly increases the incidences of apoptosis and autophagy. However, we cannot say yet to what extent this contributes to epithelial damage. Caspase inhibition experiments will be important in the future to determine this. Importantly, we can exclude that epithelial necrosis would contribute unspecifically to barrier disruption. Additionally, signalling interaction between Gal/GalNAc lectin and glycosylated receptors on the epithelial surface may cause *E. histolytica*-induced host cell death [50]. It remains to be demonstrated whether EhCPADH112 also participates in amoeba-induced barrier disruption mediated by apoptosis and autophagy.

In conclusion, our findings reveal novel initial molecular steps that facilitate *E. histolytica* entrance into the epithelial barrier. We propose that barrier disruption is likely mediated by specific interactions between EhCPADH112 and the TJ molecules occludin and claudin-1, and subsequent degradation of occludin, claudin-1, ZO-1 and ZO-2. Further experiments are necessary to prove the precise role of EhCPADH112 and other molecules in the entire virulence process. Cellular location of relevant parasite proteins in target cells, identification of their target cell receptors, as well as functional alterations of target cells are fundamental events that need to be unravelled in the future to the improve our understanding of amoebiasis.

## Supporting Information

Figure S1 ***Entamoeba histolytica*** **trophozoites cause barrier disruption in a dose-dependent manner.** MDCK **(A)** and Caco-2 **(B)** monolayers were incubated with different amounts of T for 1 h and 2 h, respectively. TER was evaluated and normalized to the TER obtained before treatment for each transwell (∼2,220 Ω·cm^2^). Mean and standard error for each time point are displayed (n = 3).
(TIF)

Figure S2 **EhCP112 and EhADH112 co-localize with occludin at TJs.** MDCK monolayers were left untreated (0 min) or incubated with TE for 2 or 30 min. **A)** Epithelial cells were processed for immunofluorescence experiments and localization of occludin (red) and EhCP112 (green) was detected with specific antibodies. **B)** Immunofluorescence assays to determine the localization of occludin (green) and EhADH112 (red). Left panels: phase contrast images of MDCK cells. Arrows: co-localization at cellular borders. Empty arrowheads: separate localization of occludin and EhCP112 or EhADH112, respectively. *Zy*-planes: co-localization at TJs (full arrowheads). Bars = 10 µm.
(TIF)

Figure S3 **TE barely affect the actin cytoskeleton.** MDCK cells were left untreated (0 min) or treated for 30 min with TE and processed for immunofluorescence assays. Localization of EhCPADH112 and actin filaments was determined using mαEhCPADH112 antibody (green) and TRITC-phalloidin (red), respectively. Nuclei were stained with DAPI. Left panels: phase contrast images of MDCK cells. Arrows: co-localization at cell borders. *Zy*-planes: co-localization at lateral membranes (full arrowheads). Bar = 10 µm.
(TIF)

Methods S1 **Supporting materials and methods.**
(DOC)
