# Supplementary figures and images for: The EhCPADH112 Complex of Entamoeba histolytica Interacts with Tight Junction Proteins Occludin and Claudin-1 to Produce Epithelial Damage
Source: PLoS One. 2013 Jun 7;8(6):e65100. doi: 10.1371/journal.pone.0065100 (PMC3676397; doi:10.1371/journal.pone.0065100)

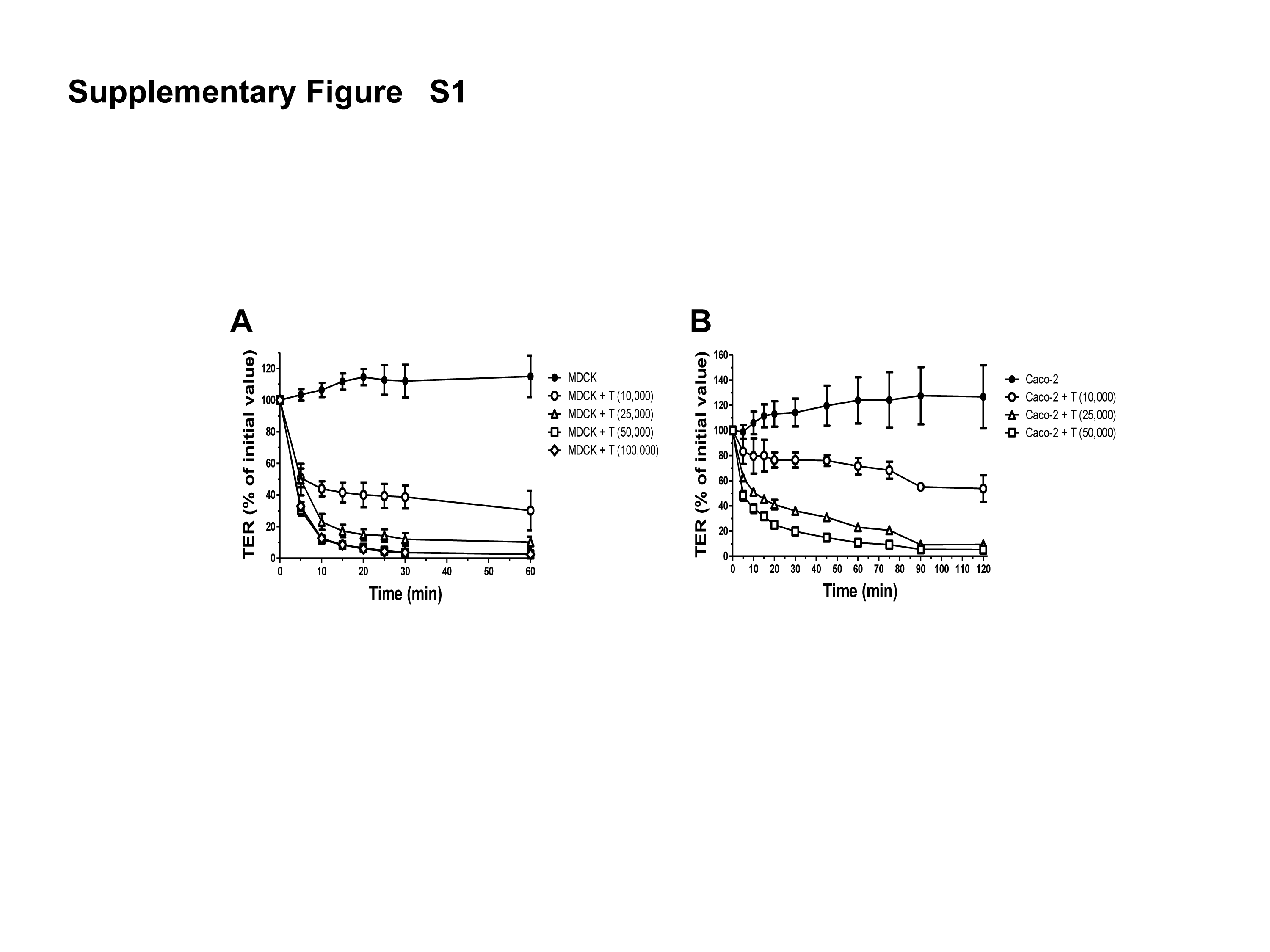

Supplement: Figure S1 — Entamoeba histolytica trophozoites cause barrier disruption in a dose-dependent manner. MDCK (A) and Caco-2 (B) monolayers were incubated with different amounts of T for 1 h and 2 h, respectively. TER was evaluated and normalized to the TER obtained before treatment for each transwell (∼2,220 Ω·cm2). Mean and standard error for each time point are displayed (n = 3). (TIF) [file pone.0065100.s001.tif]

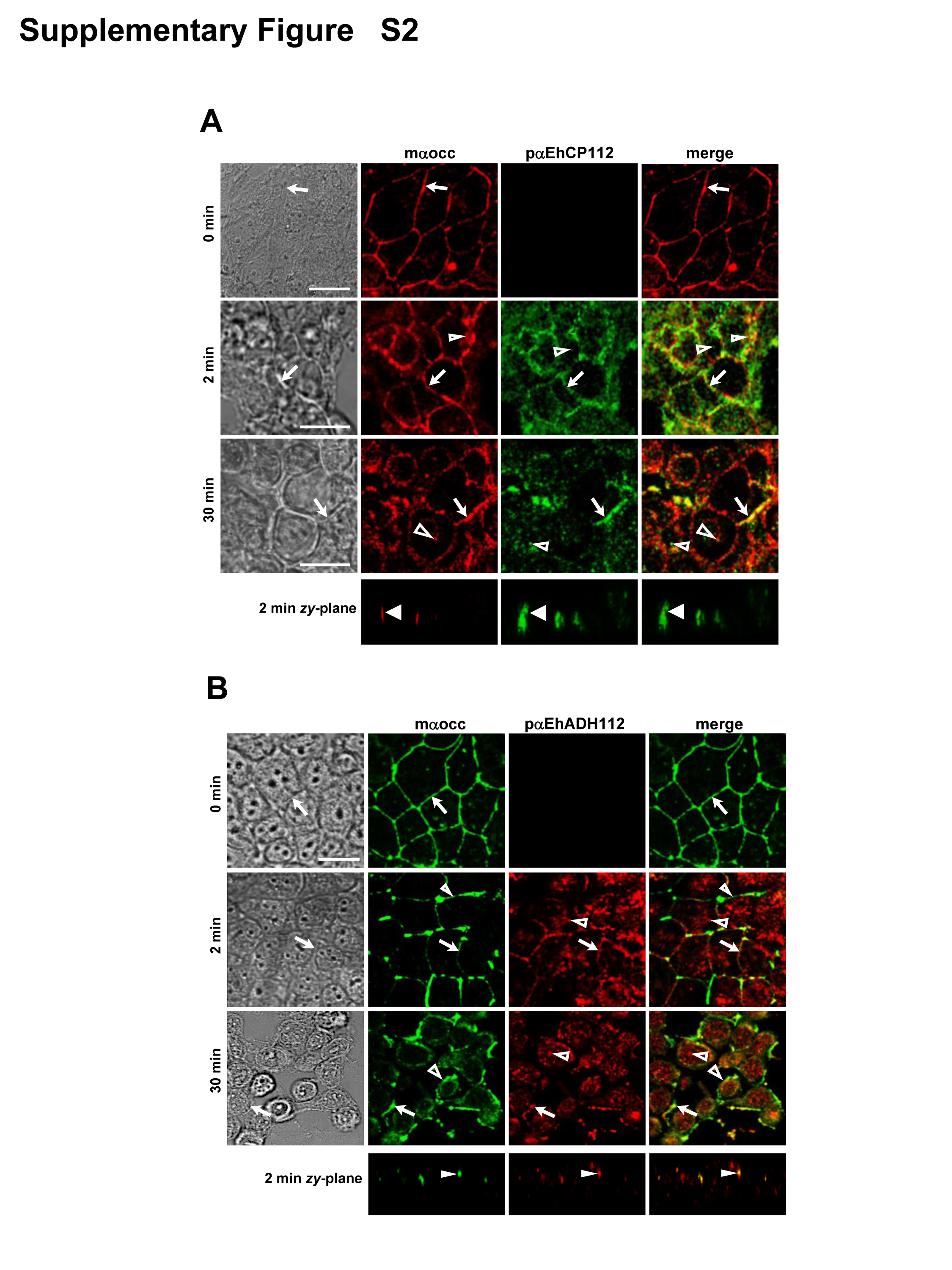

Supplement: Figure S2 — EhCP112 and EhADH112 co-localize with occludin at TJs. MDCK monolayers were left untreated (0 min) or incubated with TE for 2 or 30 min. A) Epithelial cells were processed for immunofluorescence experiments and localization of occludin (red) and EhCP112 (green) was detected with specific antibodies. B) Immunofluorescence assays to determine the localization of occludin (green) and EhADH112 (red). Left panels: phase contrast images of MDCK cells. Arrows: co-localization at cellular borders. Empty arrowheads: separate localization of occludin and EhCP112 or EhADH112, respectively. Zy-planes: co-localization at TJs (full arrowheads). Bars = 10 µm. (TIF) [file pone.0065100.s002.tif]

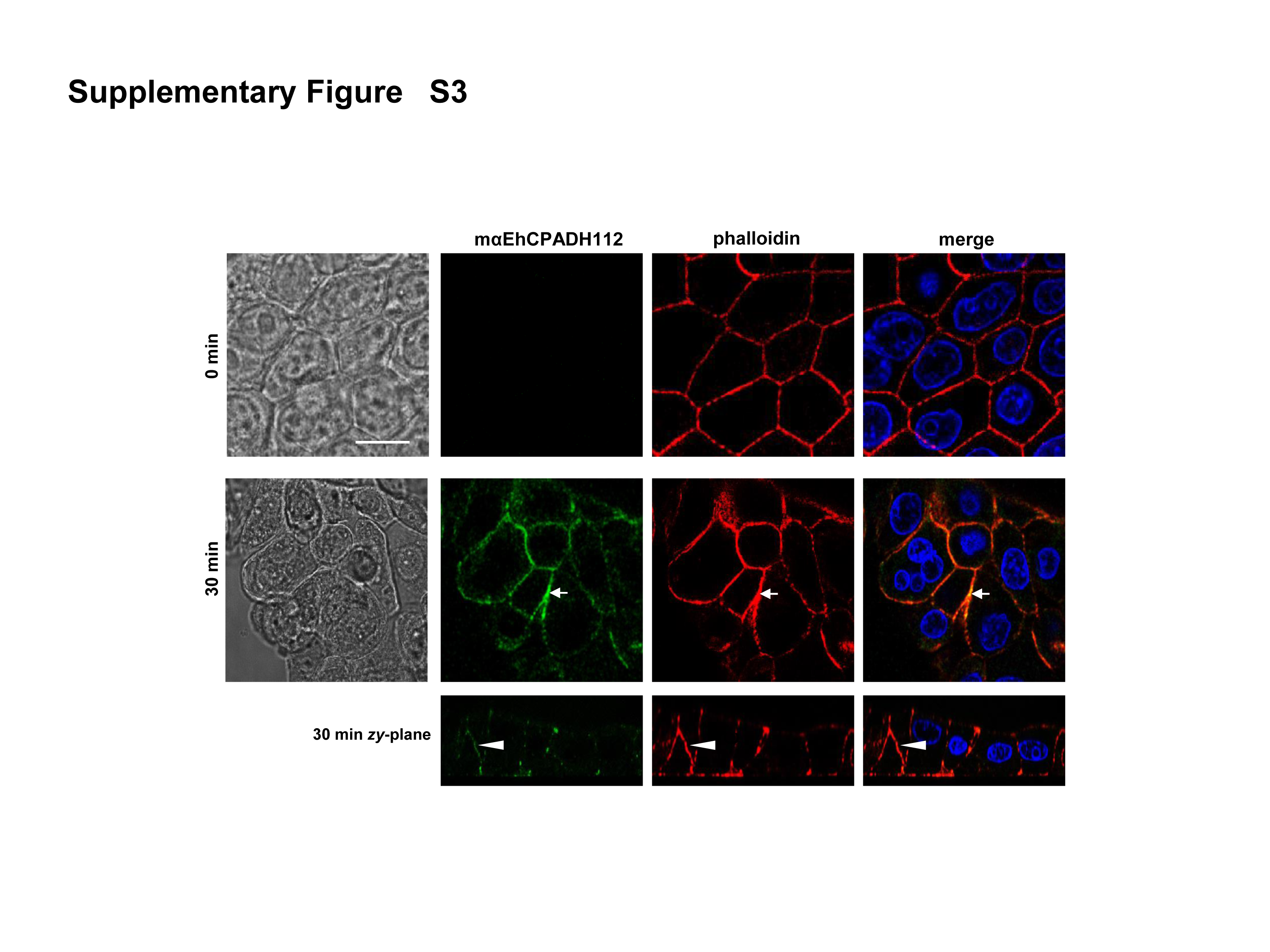

Supplement: Figure S3 — TE barely affect the actin cytoskeleton. MDCK cells were left untreated (0 min) or treated for 30 min with TE and processed for immunofluorescence assays. Localization of EhCPADH112 and actin filaments was determined using mαEhCPADH112 antibody (green) and TRITC-phalloidin (red), respectively. Nuclei were stained with DAPI. Left panels: phase contrast images of MDCK cells. Arrows: co-localization at cell borders. Zy-planes: co-localization at lateral membranes (full arrowheads). Bar = 10 µm. (TIF) [file pone.0065100.s003.tif]
