# Supplementary material for: The EhCPADH112 Complex of Entamoeba histolytica Interacts with Tight Junction Proteins Occludin and Claudin-1 to Produce Epithelial Damage
Source: PLoS One. 2013 Jun 7;8(6):e65100. doi: 10.1371/journal.pone.0065100 (PMC3676397; doi:10.1371/journal.pone.0065100)
Supplement: Methods S1 — Supporting materials and methods. (DOC) [file pone.0065100.s004.doc]

**SUPPLEMENTARY MATERIALS AND METHODS**

**Transepithelial electrical resistance (TER):** Confluent MDCK and Caco-2 cells were grown on transwell filters (0.04 µm, Corning) and incubated for 2 h with TE from 10, 25, 50 and 100 x 10^3^ trophozoites. TER values were obtained using an EVOM epithelial voltohmmeter (World Precision Instruments) and each measurement was normalized to the TER obtained before treatment (n=3).

**Immunofluorescence assays:** MDCK cell monolayers incubated with TE were fixed and permeabilized with 96% ethanol for 30 min at -20°C. Then, cells were blocked with 0.5% BSA and 0.03% saponin and incubated overnight with a mixture of mαocc and pαEhCP112 or pαEhADH112 antibodies. Cells were washed and incubated with species-specific FITC-labelled or TRITC-labelled secondary antibodies. In some experiments, actin was stained with TRITC-phalloidin and nuclei were labelled with DAPI for 5 min. After washing, cells were mounted using Vectashield (Vector) [32] and preparations were analysed by laser confocal microscopy (Leica TCS_SP5_MO).
